# VIG-1 is required for maintenance of genome stability in *Caenorhabditis elegans*

**DOI:** 10.1080/19768354.2018.1476410

**Published:** 2018-05-24

**Authors:** Bala Murali Krishna Vasamsetti, Yang-Seo Park, Nam Jeong Cho

**Affiliations:** Department of Biochemistry, College of Natural Sciences, Chungbuk National University, Cheongju, South Korea

**Keywords:** *Caenorhabditis elegans*, chromosome segregation, DNA damage response, genome stability, VIG-1

## Abstract

To explore the function of VIG-1 in *Caenorhabditis elegans*, we analyzed the phenotypes of two *vig-1* deletion mutants: *vig-1*(*tm3383*) and *vig-1*(*ok2536*). Both *vig-1* mutants exhibited phenotypes associated with genome instability, such as a high incidence of males (Him) and increased embryonic lethality. These phenotypes became more evident in succeeding generations, implying that the germline of *vig-1* accumulates DNA damage over generations. To examine whether *vig-1* causes a defect in the DNA damage response, we treated worms with UV or camptothecin, a specific topoisomerase I inhibitor. We observed that the embryonic survival of the *vig-1* mutants was reduced compared with that of the wild-type worms. Our results thus suggest that VIG-1 is required for maintaining genome stability in response to endogenous and exogenous genotoxic stresses.

## 1. Introduction

Genome integrity is critical for the survival and reproduction of living organisms. The genome is constantly damaged by diverse chemical and physical agents from endogenous and exogenous sources. Thus, cells have evolved intricate mechanisms, collectively termed the DNA damage response (DDR), to overcome the genotoxic stresses that disturb genome integrity. The DDR detects DNA damage and evokes appropriate cellular processes (Ciccia and Elledge 2010). The failure of the DDR to preserve genome integrity often results in diseases, including cancers and neurodegenerative disorders (Jackson and Bartek 2009; O'Driscoll 2012).

The nematode *Caenorhabditis elegans* (*C. elegans*) is a good model for studying the DDR mechanisms (Stergiou and Hengartner 2004; Rose 2014). Genes responsible for the DDR can be readily identified because the corresponding mutants frequently show increased embryonic lethality and/or a high incidence of males (Him) as phenotypes. As most of the genes involved in the DDR are conserved between worms and mammals, studies of *C. elegans* could provide valuable information for understanding the human DDR and for treating relevant human diseases.

*C. elegans* germline is a lineage of immortal cells that are specialized to produce gametes (Smelick and Ahmed 2005). Defects in the DDR system often make germ cells mortal in a generation-dependent fashion, and the animals with these defects eventually become sterile. For example, animals carrying a loss-of-function mutation in the *mrt-2* gene, which encodes a checkpoint protein for the DDR, reproduce normally in early generations, but progressively lose their fertility in later generations (Ahmed and Hodgkin 2000).

Caudy et al. (2003) reported that VIG-1 is a putative component of the RNA-induced silencing complex (RISC) in *C. elegans* and that depletion of VIG-1 by RNAi disrupts the *let-7* miRNA function. Recently, it has been reported that a *vig-1* deletion leads to the reduction of several miRNA species, including *let-7* (Wang et al. 2017). However, the biological function of VIG-1 is still poorly understood. In this study, we provide evidence that VIG-1 is involved in the maintenance of genome stability.

## 2. Materials and methods

### 2.1. C. elegans strains and maintenance

The strains used in this study were Bristol N2 (wild type), FX03383 *vig-1*(*tm3383*), and RB1933 *vig-1*(*ok2536*). FX03383 *vig-1*(*tm3383*) was obtained from Shohei Mitani, and RB1933 *vig-1*(*ok2536*) was obtained from the *C. elegans* Genetics Center. Worms were cultivated using standard methods (Brenner 1974). All strains were maintained at 20°C unless otherwise stated. Before initiating experiments, the *vig-1* mutants were outcrossed to wild-type worms for six times.

### 2.2. Phenotypic analyses

Worms were cultured at 20°C for various generations, and single L4 hermaphrodites of each generation were placed on NGM plates seeded with *E. coli* OP50. They were allowed to lay eggs at 20 or 25°C, and further experiments were carried out at the constant temperature of 20 or 25°C. Worms were transferred to fresh plates every 24 h until the worms no longer laid eggs.

#### 2.2.1. Eggs laid per worm

The number of eggs laid by each worm was counted while transferring the worms to fresh NGM plates seeded with OP50.

#### 2.2.2. Embryonic lethality

The number of unhatched eggs was counted 24 h after the eggs were laid. The embryonic lethality (%) was determined by counting the number of unhatched eggs per 100 eggs laid.

#### 2.2.3. Male ratio

The males were counted when the hatched progeny reached the adult stage. The adult male can be distinguished from the hermaphrodite by its small size and unique tail shape. The male ratio (%) was determined by counting the number of males per 100 adult worms.

### 2.3. Hoechst staining

To observe the chromosomes at the diakinesis oocytes, adult worms were placed in a drop of M9 buffer on a slide, fixed with 95% ethanol for 3-5 min, treated with 76 µM Hoechst 33342 (Sigma), and immediately mounted in Prolong Gold antifade reagent (Molecular Probes). Microscopy images were captured using a fluorescence microscope (LSM 710, Carl Zeiss). For quantification of chromosomes, two diakinesis oocytes, which are close to the anterior spermatheca, were analyzed.

### 2.4. UV sensitivity assay

UV sensitivity assays were performed as described by Craig et al. (2012) with minor modifications. Synchronized L4 hermaphrodites were irradiated with various doses of UV at an emission peak of 254 nm (GS gene linker UV chamber, Bio-Rad). The UV-irradiated worms were transferred to fresh NGM plates seeded with OP50 and grown at 20°C. The eggs laid during 20-40 h following UV exposure were collected. The number of hatched worms was counted 24 h after the eggs were laid. The embryonic survival (%) was determined by counting the number of hatched animals per 100 eggs laid. The embryonic survival of the UV-irradiated worms was normalized to that of the nonirradiated control worms.

### 2.5. Camptothecin (CPT) sensitivity assay

CPT sensitivity assay was performed as described by Saito et al. (2012) with minor modifications. Synchronized L4 hermaphrodites were grown on NGM plates seeded with OP50 for 19 h at 20°C. The worms were then treated with various concentrations of CPT (Sigma) in M9 buffer containing OP50 with gentle shaking in the dark for 19 h. After the CPT-treated worms were allowed to recover for 3 h on NGM plates seeded with OP50, eggs were collected for 4 h. The number of hatched worms was counted 24 h after the eggs were laid. The embryonic survival (%) was determined by counting the number of hatched animals per 100 eggs laid. The embryonic survival of the CPT-treated worms was normalized to that of the nontreated control worms.

## 3. Results

### 3.1. vig-1 mutants show a progressive loss of fertility

**Figure 1.**
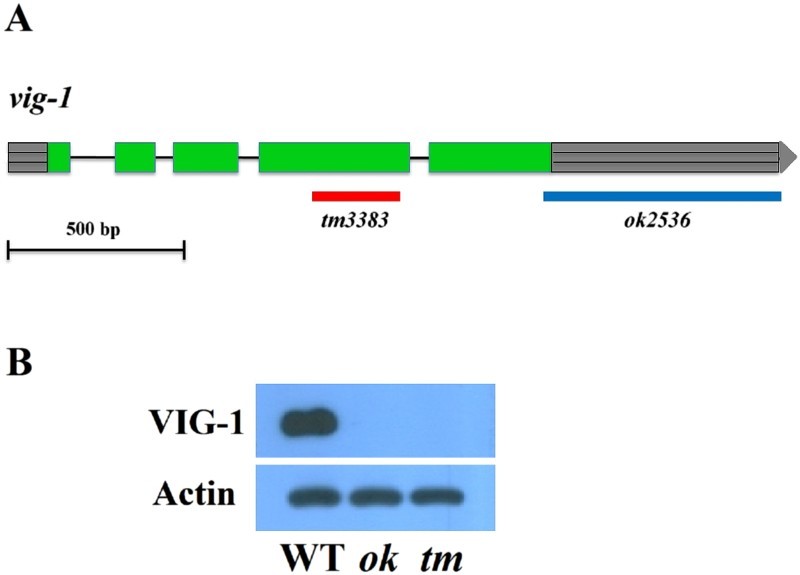
Lack of the VIG-1 protein in two *vig-1* deletion mutants. (A) Deleted DNA regions in the two *vig-1* mutants are shown. Green boxes represent exons, lines represent introns, and gray boxes with horizontal lines at both ends represent 5′ and 3′ UTRs. (B) Immunoblot analysis of VIG-1 expression. Protein lysates were prepared from mixed-stage populations of the indicated strains, and immunoblotted with a monoclonal antibody against VIG-1. Actin was used as a loading control. WT, wild type; *ok*, *vig-1(ok2536)*; *tm*, *vig-1(tm3383)*.

To explore the biological function of VIG-1, we analyzed the phenotypes of two *vig-1* deletion mutants: *vig-1*(*tm3383*) and *vig-1*(*ok2536)*. *vig-1*(*tm3383)* has a 250-bp deletion within the protein coding sequence, whereas *vig-1*(*ok2536)* has a 676-bp deletion, which corresponds to the protein coding sequence of the last 14 amino acids and entire predicted 3′ untranslated region (UTR) (Figure 1(A)). We tested whether these two *vig-1* mutants produce the VIG-1 protein by performing western blot experiments using an anti-VIG-1 monoclonal antibody. The antibody detected a protein band of approximately 50 kDa in the wild-type sample, but no band was found in the *vig-1* mutants (Figure 1(B)), showing that the mutants are defective in producing the VIG-1 protein.

**Figure 2.**
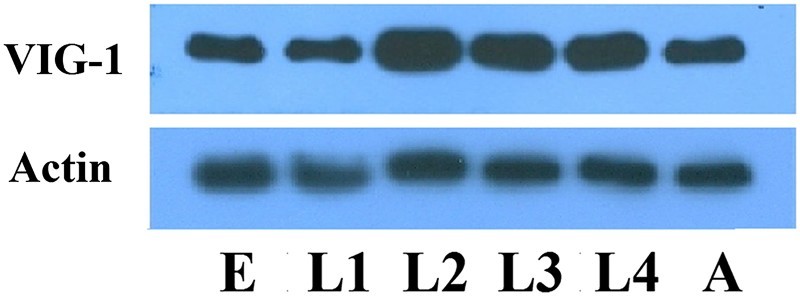
Temporal pattern of VIG-1 expression. Protein lysates were prepared from wild-type worms at each developmental stage, and immunoblotted with an anti-VIG-1 antibody. Actin was used as a loading control. E, egg stage; L1-L4, larval stages; A, adult stage.

We next determined the temporal expression pattern of VIG-1 in the wild-type animals with the anti-VIG-1 monoclonal antibody. VIG-1 expression was observed at each developmental stage examined (Figure 2), implying that VIG-1 plays a role in all the developmental stages.

**Figure 3.**
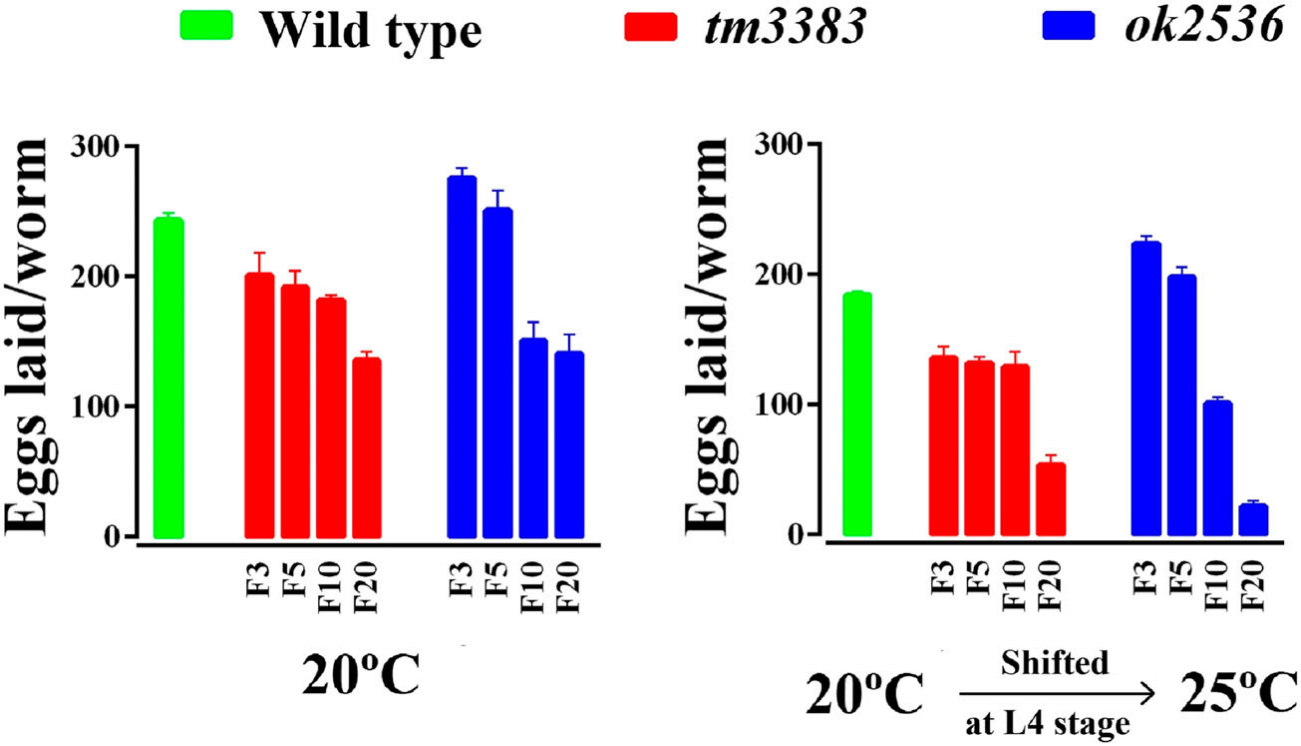
The total number of eggs laid by the *vig-1* mutants decreases over generations. Worms were cultured at 20°C for various generations, and single L4 hermaphrodites at the indicated generation were transferred to individual plates and allowed to lay eggs at either 20°C (left panel) or 25°C (right panel). The total number of eggs laid by the worms was determined as described in the Materials and methods. Error bars indicate SEM (*n* = 10).

When we analyzed the phenotypes of homozygous *vig-1* mutants (*vig-1/vig-1*) directly derived from a heterozygote (*vig-1/+*), very mild to no difference from the wild-type animals was noted at 20°C. However, the *vig-1* mutants of succeeding generations laid a smaller number of eggs than the wild-type animals at 20°C (Figure 3, left panel). In generation F3, *vig-1(tm3383)* laid 201 ± 17.4 eggs, but only 135 ± 7.0 eggs were laid at F20. In the case of *vig-1(ok2536)*, 276 ± 7.4 and 141 ± 14.7 eggs were laid at F3 and F20, respectively. By comparison, wild-type animals laid ∼250 eggs in all the generations tested.

**Figure 4.**
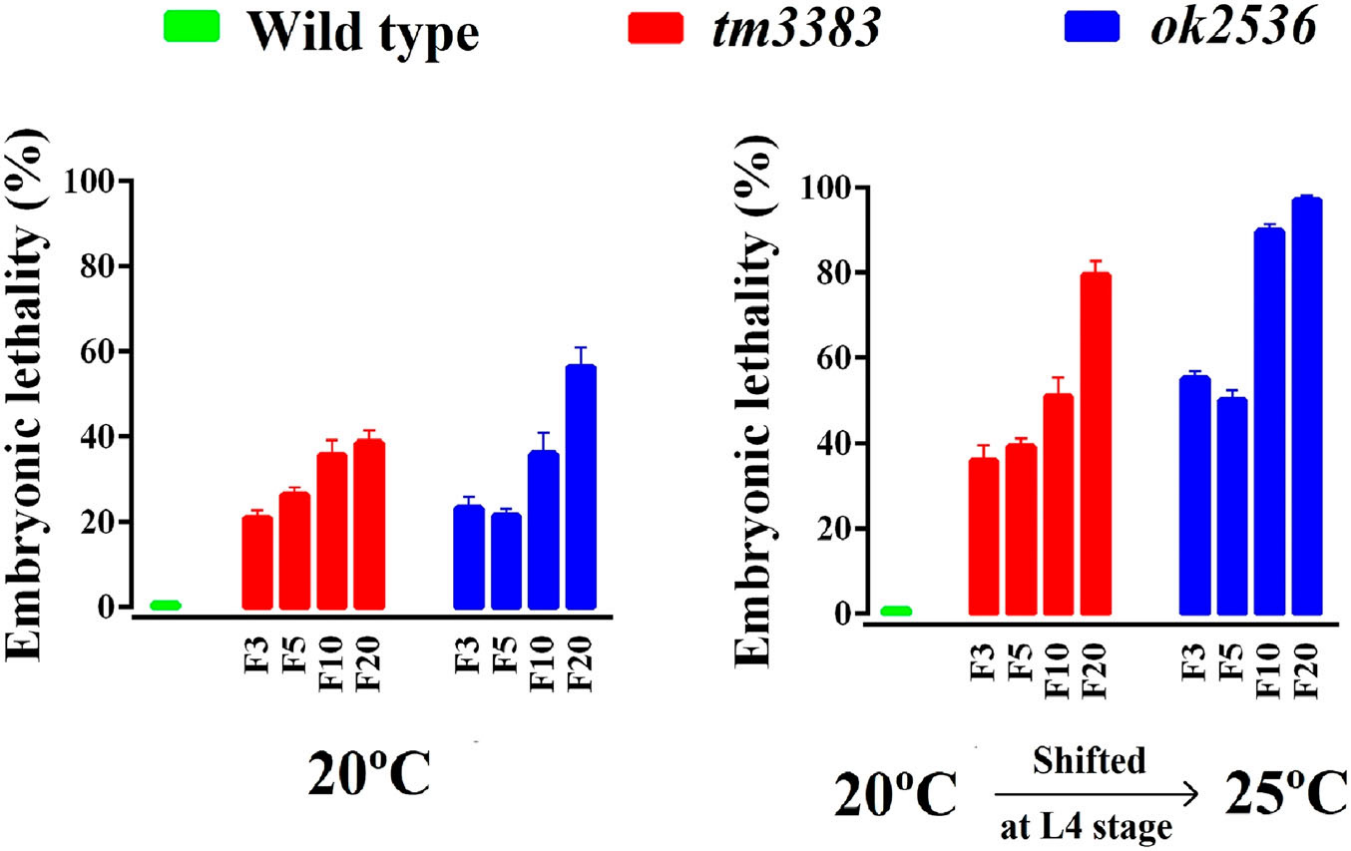
The embryonic lethality of the *vig-1* mutants increases over generations. Worms were cultured at 20°C for various generations, and single L4 hermaphrodites at the indicated generation were transferred to individual plates and allowed to lay eggs at either 20°C (left panel) or 25°C (right panel). The embryonic lethality (%) was determined as described in the Materials and methods. Error bars indicate SEM (*n* = 10).

We also observed that the *vig-1* mutants displayed higher embryonic lethality than the wild-type animals at 20°C (Figure 4, left panel). In generation F3, *vig-1* deletion caused ∼20% embryonic lethality, which gradually increased in subsequent generations. As a consequence, the *vig-1* mutants produced smaller brood sizes (total number of progeny/worm) than the wild-type animals at 20°C. For example, in generation F3, brood sizes of *vig-1(tm3383)* and *vig-1(ok2536)* were 158 ± 12.1 and 211 ± 7.0, respectively. In generation F10, brood sizes decreased to 117 ± 7.0 for *vig-1(tm3383)* and 93 ± 9.1 for *vig-1(ok2536)*. In generation F20, brood sizes were further reduced to 83 ± 6.5 for *vig-1(tm3383)* and 65 ± 12.5 for *vig-1(ok2536)*. The brood size of wild-type animals was ∼240, regardless of the generation number. This progressive decrease in brood size evoked by *vig-1* deletion suggests that the germline of *vig-1* mutants gradually accumulates genetic alterations over generations.

We also found that the hatched progeny of the *vig-1* mutants showed diverse types of morphological abnormalities, including a growth arrest, protruding vulva, and aberrant male tail structure (Supplementary Figure 1). Again, these abnormalities were less frequent in earlier generations, but were clearly visible in later generations.

As many mutations affecting genome stability are temperature-sensitive (Ahmed and Hodgkin 2000; Lee et al. 2003; Lee et al. 2004), we examined whether the *vig-1* mutants are sensitive to high temperature. When the *vig-1* mutants were shifted to 25°C during the L4 stage, the phenotypes of the *vig-1* mutants were enhanced. The number of eggs laid per worm was greatly reduced in the *vig-1* mutants, especially at F20, whereas that in the wild-type animals was moderately reduced, regardless of generation (Figure 3, right panel). Temperature-shift of the *vig-1* mutants to 25°C resulted in a further ∼2-fold increase in embryonic lethality compared with the values at 20°C in all generations tested (Figure 4, right panel). Furthermore, if the hatched progeny of the *vig-1* mutants were allowed to grow at 25°C, they often exhibited developmental abnormalities, such as a growth arrest. Although some progeny could survive and reach adulthood, they usually failed to lay eggs. This high-temperature sterility was seen even in early generations. The biochemical reason for the sensitivity of the *vig-1* mutants to high temperature is currently unknown.

### 3.2. vig-1 mutants are defective in meiotic chromosome segregation

**Figure 5.**
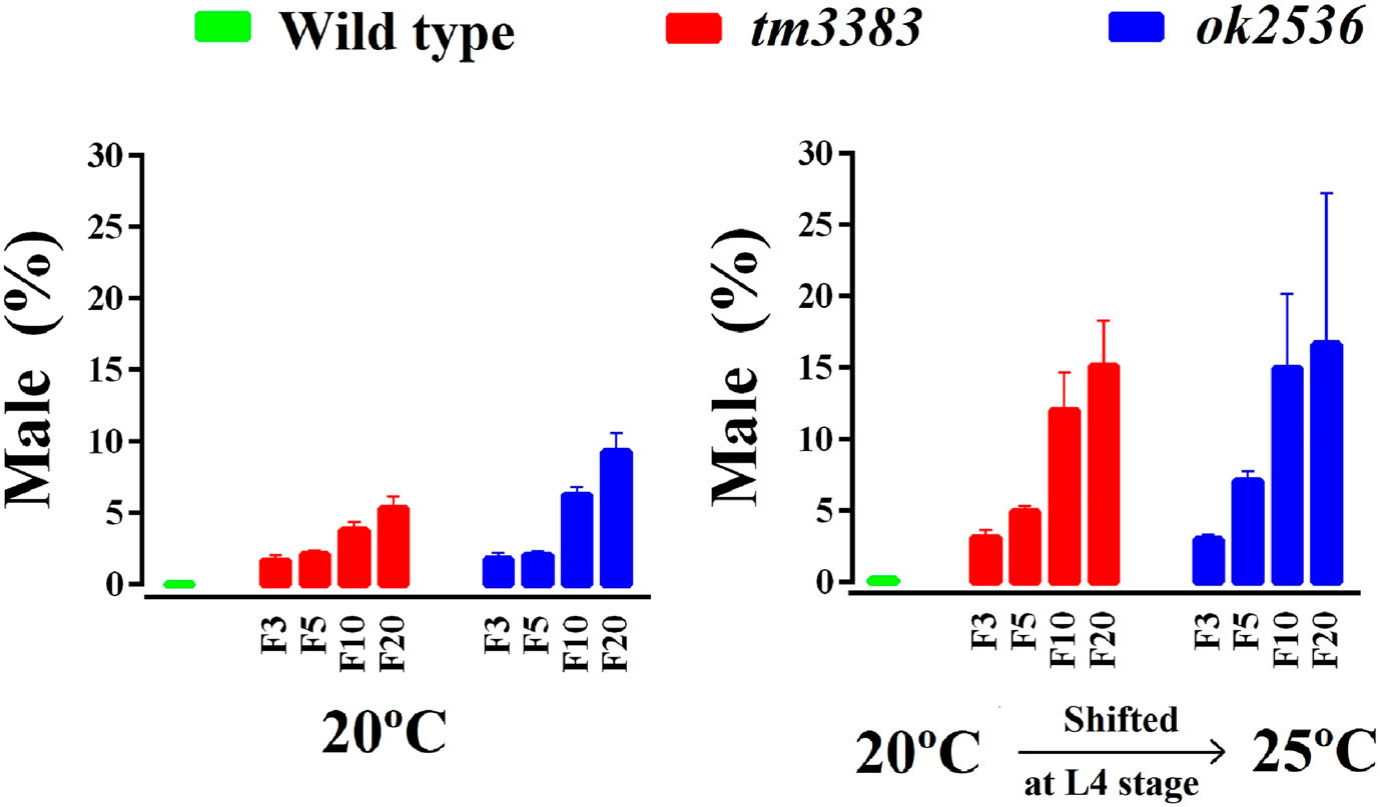
The male ratio of *vig-1* mutants increases over generations. Worms were cultured at 20°C for various generations, and single L4 hermaphrodites at the indicated generation were allowed to lay eggs at either 20°C (left panel) or 25°C (right panel). The male (%) was determined as described in the Materials and methods. Error bars indicate SEM (*n* = 10).

*C. elegans* populations are normally XX hermaphrodites (5 pairs of autosomes and 2X chromosomes), but XO males arise due to X-chromosome loss during meiosis with a frequency of ∼0.2% (Hodgkin et al. 1979). We found that both *vig-1* mutants had a high incidence of the males (Him) phenotype at 20°C (Figure 5, left panel). In generation F3, *vig-1* deletion caused ∼2% of the worms to be males. When the *vig-1* mutant worms reached F20, the male ratio increased to 5-9%, whereas wild-type worms showed less than 0.1% males. We also observed that temperature-shift of the *vig-1* mutants to 25°C markedly increased the male ratio (Figure 5, right panel). These data indicate that X-chromosome nondisjunction is promoted due to the lack of VIG-1. Conceivably, the VIG-1 protein is necessary for proper meiotic chromosome segregation.

**Figure 6.**
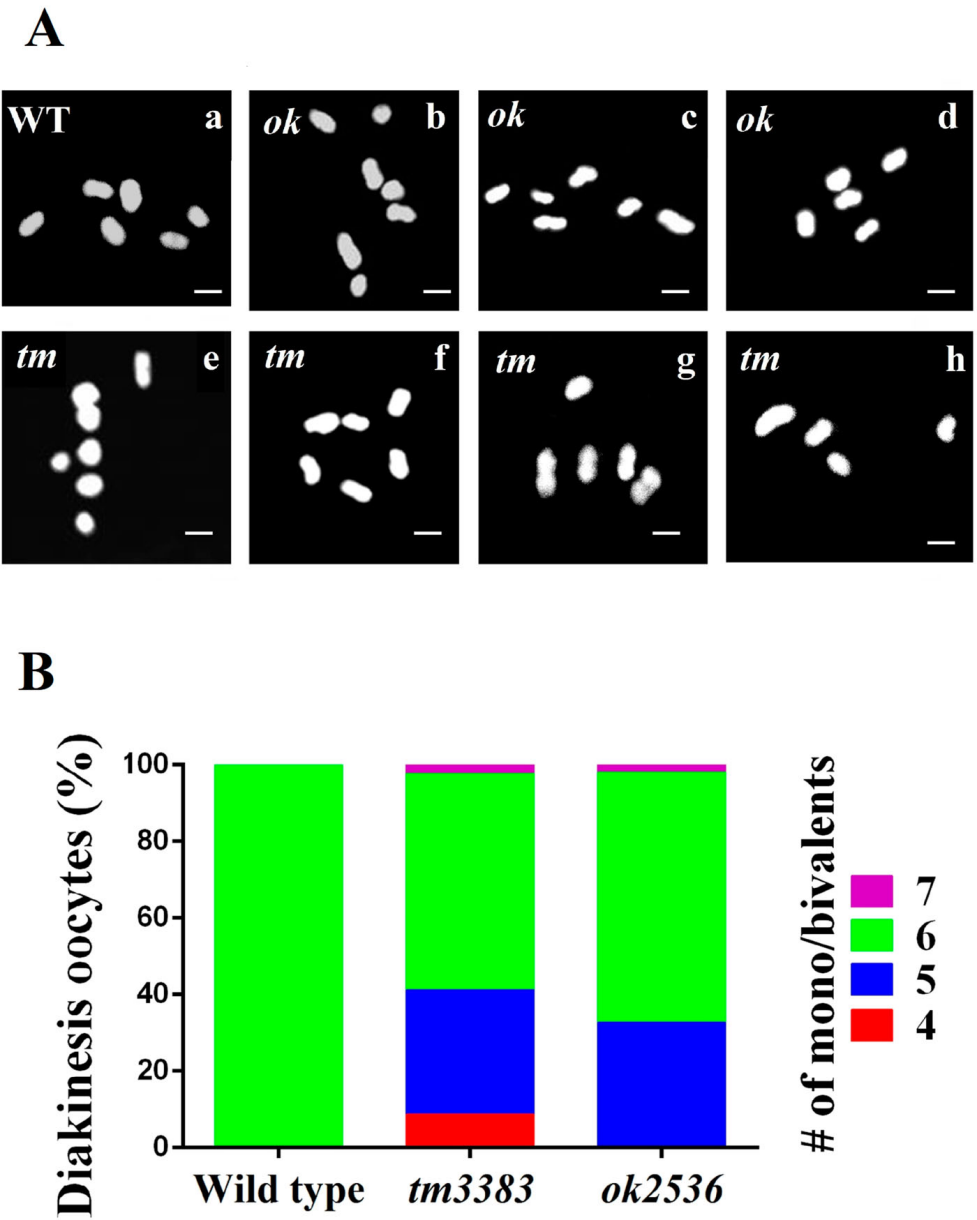
*vig-1* deletion causes defects in chromosomal segregation during meiosis. (A) *vig-1* deletion induces chromosomal aberrations in the diakinesis oocytes. F3 generation adult hermaphrodites were stained with Hoechst dye, and the chromosomes in the diakinesis oocytes were observed using a fluorescence microscope. Whereas all the wild-type animals (WT) examined had normal 6 chromosomal bivalents (a), the *vig-1* mutants exhibited 4-7 chromosomal bodies (b-h). The *vig-1* mutants with 7 mono/bivalents (b and e), 6 mono/bivalents (c and f), 5 mono/bivalents (d and g), and 4 mono/bivalents (h) are shown. *ok*, *vig-1(ok2536)*; *tm*, *vig-1(tm3383)*. Scale bar, 2 µm. (B) Chromosomal abnormalities seen in F3 generation *vig-1* mutants are quantified.

As a high incidence of the males (Him) phenotype was evident in the *vig-1* mutants, we asked if *vig-1* induces chromosomal alterations in the diakinesis oocytes. During diakinesis, which is the last stage of meiotic prophase I, *C. elegans* chromosomes are highly condensed and easily identified by staining DNA (Hillers et al. 2017). When the wild-type hermaphrodites were treated with Hoechst dye, typical 6 bivalents were observed; however, various abnormalities in chromosome number and size were detected in the *vig-1* mutants (Figure 6(A)). Some diakinesis oocytes of the *vig-1* mutants had more than six Hoechst-stained bodies, and many other diakinesis oocytes possessed less than six bodies. In generation F3, the frequencies of the diakinesis oocytes with aberrant chromosome structure were 43.5% (20 out of 46) for *vig-1(tm3383)* and 34.6% (18 out of 52) for *vig-1(ok2536)* (Figure 6(B)). Overall, our results strongly suggest that VIG-1 serves an important function in meiotic processes.

### 3.3. vig-1 mutants are sensitive to exogenous DNA damage

**Figure 7.**
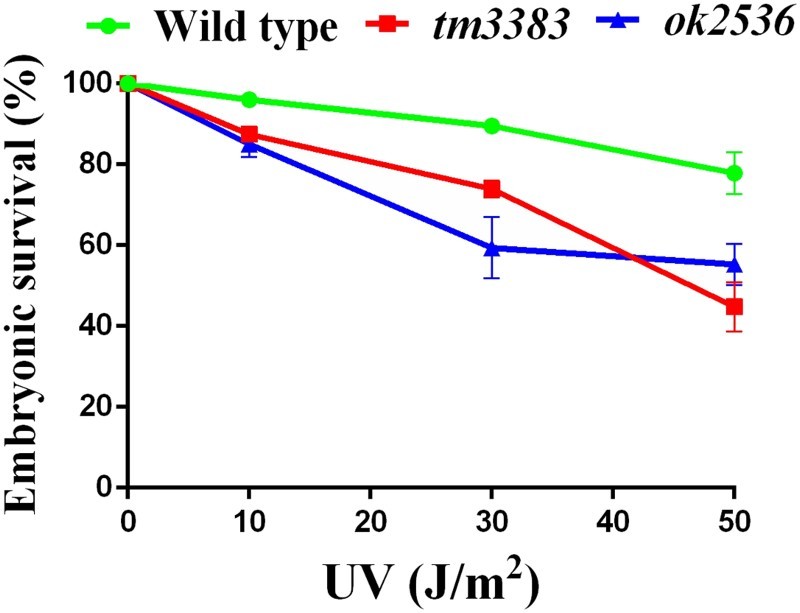
The *vig-1* mutants are sensitive to UV. F3 generation L4 hermaphrodites were irradiated with various doses of UV, and the embryonic survival (%) was determined as described in the Materials and methods. More than 100 embryos were used for each data point. Similar results were obtained from three separate experiments, and error bars indicate SEM.

Like other living organisms, *C. elegans* possesses elaborate DDR mechanisms to protect the genome from genotoxic stresses (Stergiou and Hengartner 2004). In fact, *C. elegans* mutants with a defect in the DDR exhibit decreased embryonic survival in response to DNA-damaging agents, such as UV radiation (Park et al. 2004) and camptothecin (CPT) treatment (Hyun et al. 2016). To investigate whether the *vig-1* mutants are defective in eliciting the DDR, we subjected L4-stage *vig-1* mutant worms to UV radiation. Although the embryonic survivals of both the wild-type worms and *vig-1* mutants were reduced in a dose-dependent manner, the *vig-1* mutants were more sensitive to UV exposure than the wild-type worms (Figure 7). In response to 50 J/m^2^ UV, *vig-1*(*tm3383*), *vig-1*(*ok2583*), and wild-type worms showed 45 ± 6.5%, 55 ± 5.1%, and 78 ± 5.2% embryonic survival, respectively. This hypersensitivity of the *vig-1* mutants implies that VIG-1 participates in the DDR process against UV.

**Figure 8.**
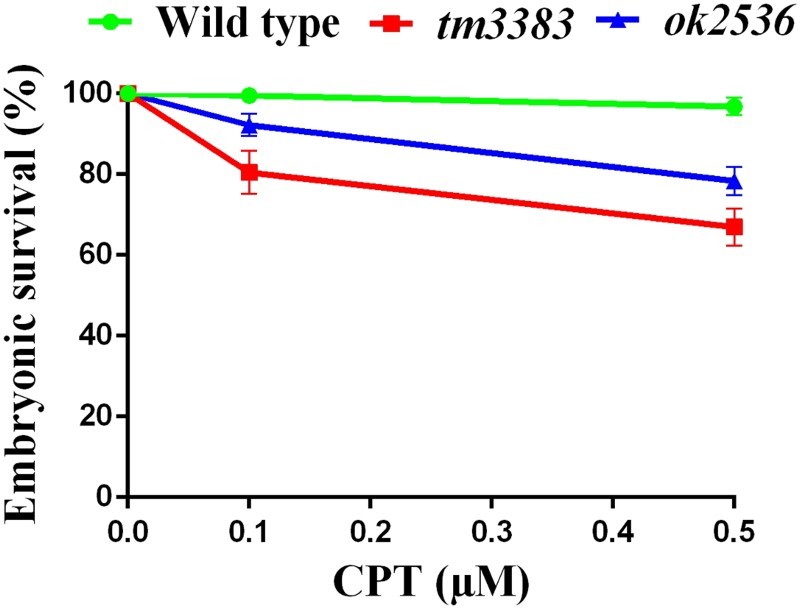
The *vig-1* mutants are sensitive to CPT. F3 generation young adults were treated with 0.1 or 0.5 μM CPT for 19 h, and the embryonic survival (%) was determined as described in the Materials and methods. More than 100 embryos were used for each data point. Similar results were obtained from four separate experiments, and error bars indicate SEM.

Double strand breaks (DSBs) are generated by DNA damage signals from either physiological (endogenous) or environmental (exogenous) sources, and cells activate the homologous recombination (HR) or non-homologous end joining (NHEJ) pathway to repair DSBs (Ciccia and Elledge 2010; Rastogi et al. 2010). To test the possibility that VIG-1 plays a role in DSB repair, we treated the worms with CPT, a specific topoisomerase I inhibitor, which makes DSBs at replication forks (Liu et al. 2000; Sakasai and Iwabuchi 2015). We observed that the embryonic survival of the *vig-1* mutants was substantially diminished compared with that of the wild-type worms (Figure 8). Upon the exposure to 0.5 µM CPT, *vig-1*(*tm3383*) and *vig-1*(*ok2583*) showed 67 ± 4.6% and 78 ± 3.5% embryonic survival, respectively. By comparison, the embryonic survival of wild-type worms was 97 ± 2.1% with 0.5 µM CPT treatment. These results support the notion that VIG-1 is involved in the CPT-induced DSB repair.

## 4. Discussion

Several lines of evidence described in this study indicate that VIG-1 is a regulator of genome stability in *C. elegans*. First, *vig-1* mutants exhibited a progressive loss of fertility. Second, *vig-1* mutants revealed aberrant chromosome segregation during meiosis. Third, *vig-1* mutants were more sensitive to DNA-damaging agents (UV and CPT) than wild-type worms.

The high degree of embryonic lethality seen in *vig-1* mutants is likely due to meiotic chromosome missegregation because embryos with a loss of autosome usually fail to hatch. It is also possible that a defective DDR system for endogenous DNA damage contributes to embryonic lethality. For instance, nonviable embryos can be produced if SPO-11-induced DSBs, which trigger meiotic recombination, are not properly repaired (Alpi et al. 2003; Penkner et al. 2007; Lemmens et al. 2013).

UV induces a wide variety of DNA lesions, such as cyclobutane pyrimidine dimers, 6-4 photoproducts, and DNA strand breaks (Rastogi et al. 2010). UV-induced DNA alterations can be repaired via diverse processes, including the nucleotide excision repair (NER), HR, and NHEJ pathways. The hypersensitivity of *vig-1* mutants to UV raises the possibility that VIG-1 serves as a component of the molecular machinery that participates in the repair of DNA damage. CPT, a specific inhibitor of topoisomerase I, generates DSBs during S phase when DNA is being replicated (Hsiang et al. 1989; Ryan et al. 1991; Sakasai and Iwabuchi 2015). The hypersensitivity of *vig-1* mutants to CPT implies that VIG-1 might play a role in DSB repair.

VIG-1 contains the RGG/RG motif, which is found in many RNA-binding proteins and shown to affect various aspects of RNA metabolism (Thandapani et al. 2013). In addition to its function in RNA binding, the RGG/RG motif provides the site for protein interaction. It thus can be postulated that VIG-1 modulates genome stability by binding to RNA molecules and/or proteins. Of note, several proteins with the RGG/RG motif (e.g. MRE11 and 53BP1) have roles in the maintenance of genome stability (Thandapani et al. 2013).

Sequence comparison indicates that VIG-1 is homologous to human SERPINE1 mRNA binding protein 1 (SERBP1, also called PAI-RBP1). Recently, Ahn et al. (2015) reported that SERBP1 influences the DDR by promoting the translation of CtIP, a key regulator of the HR pathway (You and Bailis 2010). Deletion analysis revealed that a RGG-rich region of SERBP1 is necessary for CtIP mRNA binding (Ahn et al. 2015). It will be of interest to see if VIG-1 controls the level of the *C. elegans* homolog of CtIP, COM-1, which blocks the NHEJ pathway and facilitates the HR-mediated DSB repair during meiosis (Lemmens et al. 2013).

VIG-1 is considered to be a component of the RISC of *C. elegans* (Caudy et al. 2003). Knockdown of the *vig-1* gene interferes with the *let-7* miRNA function, suggesting that VIG-1 modulates the miRNA pathway. The role of VIG-1 in miRNA function is also supported by the observation that GLD-1, a genetic interactor of VIG-1, is required for the functions of *let-7* and *mir-35* miRNA families (Akay et al. 2013). Interestingly, accumulating evidence indicates that miRNAs play essential roles in the DDR (Landau and Slack 2011). For example, *C. elegans* miR-34 expression is upregulated in response to radiation, and the *mir-34* mutant exhibits altered sensitivity to radiation (Kato et al. 2009). It remains to be determined whether VIG-1 participates in the DDR by modulating the miRNA function.

In conclusion, our data show that VIG-1 is involved in the maintenance of genome stability by protecting DNA from endogenous and exogenous genotoxic stresses. Elucidating the molecular mechanism by which VIG-1 performs its function(s) will help us to better understand how the DDR system works in metazoans.

## Supplementary Material

SUPPORTING_INFORMATION.docx
