# Supplementary material for: VIG-1 is required for maintenance of genome stability in Caenorhabditis elegans
Source: Anim Cells Syst (Seoul). 2018 May 24;22(3):197–204. doi: 10.1080/19768354.2018.1476410 (PMC6138327; doi:10.1080/19768354.2018.1476410)
Supplement: SUPPORTING_INFORMATION.docx [file TACS_A_1476410_SM2090.docx]

**Supplementary information**

**
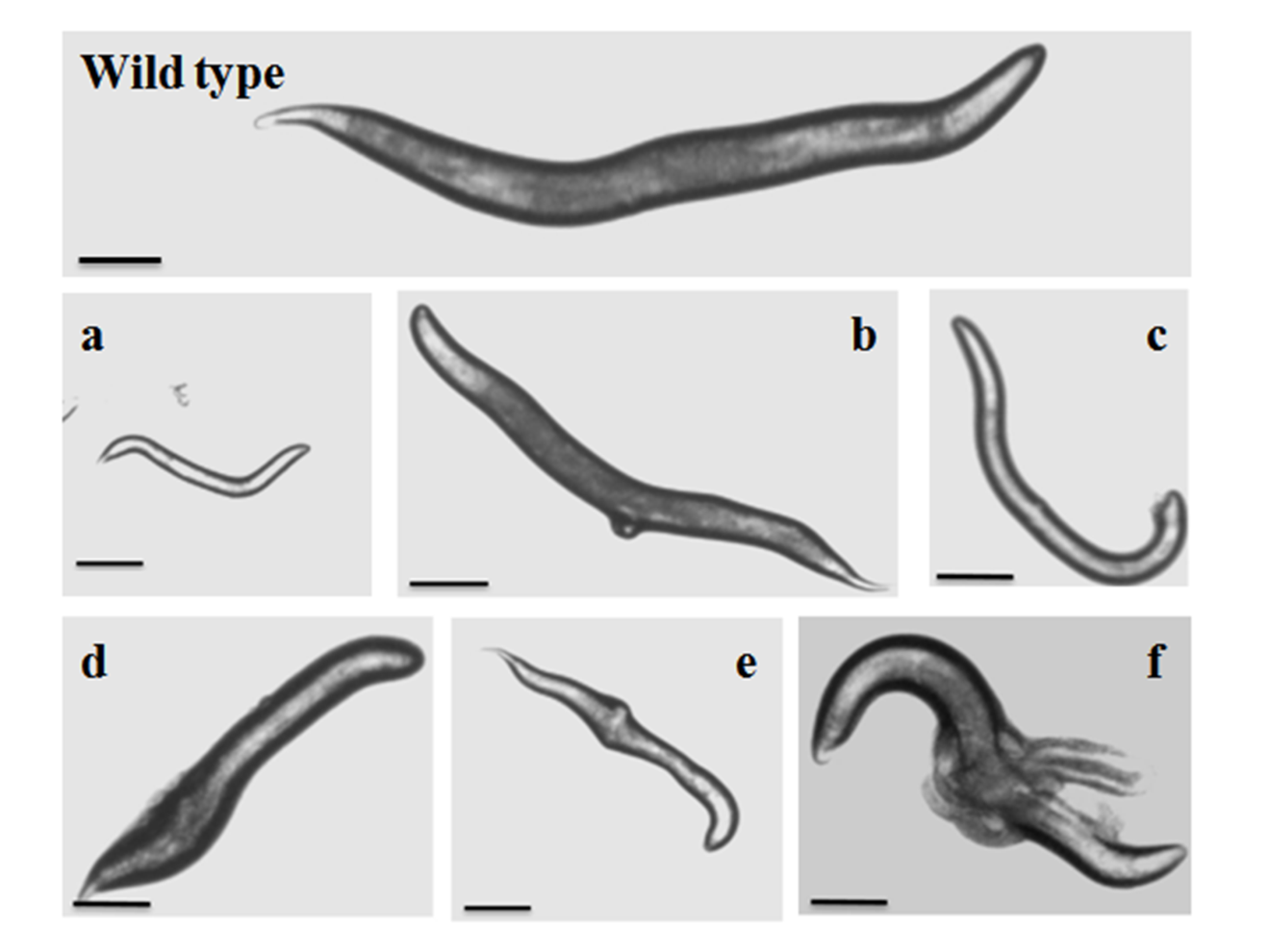
**

Figure 1. *vig-1* mutants display diverse types of morphological abnormalities during development. (a-f) Morphologically abnormal *vig-1* mutant worms were collected, and captured using a microscope (Leica DMi 8). (a) growth arrest, (b) protruding vulva, (c) aberrant male tail, (d) bulged tail region, (e) extruding bumps, and (f) ruptured body. Scale bar, 100 µm.
